# The immune response to human cytomegalovirus: impact of age, co-morbidities and the significance of anti-viral activity assessment

**DOI:** 10.1098/rstb.2024.0408

**Published:** 2025-11-06

**Authors:** Sarah Elizabeth Jackson, Mahlaqua Noor, Eleanor Lim, Mark Wills

**Affiliations:** ^1^ Medicine and CITIID, University of Cambridge School of Clinical Medicine, Cambridge CB2 0QQ, UK

**Keywords:** NK-cell, T-cells, immunosenescence, anti-viral, antibody, immune-evasion

## Abstract

Understanding how all the components of the immune system respond to human cytomegalovirus (HCMV) primary infection and subsequent reactivation events is necessary to be able to successfully treat patients suffering from HCMV-mediated disease. In the first part of this review, we overview the humoral response to HCMV, followed by sections on the response of NK cells, CD8^+^ T cells, CD4^+^ T cells and the growing field of unconventional T cell subsets to HCMV infection. We then discuss how our knowledge of the CMV-specific immune response can help to inform studies on the effects of HCMV infection in ageing and immunocompromised populations. We discuss how current clinical diagnostic tests relate to the findings from the research field into the functionality of the immune response in different patient cohorts and how the use of direct anti-viral activity in autologous settings could be used to inform intervention strategies with a view to improving patient treatment and outcomes.

This article is part of the discussion meeting issue ‘The indirect effects of cytomegalovirus infection: mechanisms and consequences’.

## 1. Introduction

The substantial health burden associated with human cytomegalovirus (HCMV) infection across multiple clinical settings underscores the designation by the US National Institute of Medicine that HCMV is a pathogen for which vaccine development and novel therapeutic strategies are of the highest priority [1]. Primary HCMV infection is typically asymptomatic but establishes lifelong persistence, a characteristic feature of all herpesviruses. This is due to the virus’s ability to establish latent infection in specific cell types, leading to an overall infection rate exceeding 60% of the global population [2,3].

A critical determinant of HCMV pathogenesis is the immune status of the host [4]. In developed countries, severe disease is most commonly observed in immunosuppressed organ transplant recipients or following congenital infection [5]. Reactivation is particularly problematic in transplant patients; for instance, in haematopoietic stem cell transplantation, up to 80% of seropositive recipients will develop HCMV viremia unless treated [4]. While existing anti-viral therapies target lytic replication, even with prophylactic anti-viral use, 20−35% of transplant recipients remain at risk of HCMV infection and multi-organ disease [6]. Consequently, HCMV continues to pose a significant clinical challenge, in part due to the reactivation of latent virus, which is insufficiently controlled in immunocompromised patients. Immunosenescence, the decline of the immune response with ageing, is characterized by increased susceptibility to novel infections and increased incidence of autoimmune diseases [7]. Long-term carriage of HCMV in older people has been implicated in increasing the incidence of morbidity and mortality from cardiovascular disease [8] and in the recent COVID-19 pandemic it was observed that HCMV positivity increased the risk of hospitalization and treatment with oxygen in patients [9,10].

A comprehensive understanding of both innate and adaptive immune responses to HCMV is essential, particularly in the context of a virus that encodes a wide array of immune evasion mechanisms targeting multiple aspects of host immunity. Moreover, accurately assessing immune responses is critical for elucidating the complex and long-term interactions between HCMV and its host.

## 2. The innate and adaptive immune response to HCMV infection

### (a) Humoral response and HCMV-specific memory B cells

HCMV requires the highly conserved virion envelope glycoproteins gB and gH/gL to infect target cells. The encoded gH/gL molecules, crosslinked through disulfide bonds, are prerequisites for cellular entry and the gH/gL dimer on the HCMV cell surface exists either as a trimeric complex—comprising gH/gL/gO—or as a pentameric complex, consisting of gH/gL/UL128/UL130/UL131A. The gH/gL/gO trimer complex is sufficient for attachment to and infection of fibroblasts, whereas the pentamer complex is essential for entry into epithelial, endothelial and myeloid cells [11–15]. HCMV primary infection elicits the production of anti-HCMV antibodies capable of mediating polyfunctional responses. The humoral immune response to HCMV targets envelope glycoproteins (predominantly gB and gH), structural tegument proteins (e.g. pp65 and pp150) and non-structural proteins such as IE1 [16,17]. While the majority of antibodies target gB—accounting for up to 70% of the neutralizing antibody response [18]—recent studies have shown that trimer- and pentamer-specific antibodies act synergistically to neutralize HCMV [19]. The importance of neutralizing antibodies in reducing the risk of congenital infection has been well documented [20]. Furthermore, the administration of HCMV-specific antibodies has been associated with favourable clinical outcomes in transplant patients [21].

Given the therapeutic potential of neutralizing antibodies, a considerable research effort over many years has been made to develop vaccines and antibody-based therapies [22]. The most efficacious vaccine candidate to date, an MF59-adjuvanted gB protein subunit vaccine (gB/MF59), demonstrated 43−50% efficacy in Phase II trials, partly through the induction of neutralizing antibodies [23,24]. In addition, it has been noted that non-neutralizing antibodies are also elicited by this vaccine which are able to bind gB on the surface of infected cells as well as HCMV virions promoting phagocytosis [25]. More recently, a first-in-human trial of an mRNA-based HCMV vaccine (mRNA-1647) elicited a potent and durable HCMV-specific IgG response in seronegative donors [26]. However, no licensed HCMV vaccine is currently available (reviewed in [27]).

Although neutralizing antibodies play a critical role in protection against HCMV, absolute antibody titres and viral neutralization abilities represent incomplete correlates of immunity [28]. One possible explanation for this lack of clinical efficacy lies in the biology of viral dissemination. Transmission between individuals involves cell-free virus, which can be efficiently inhibited by neutralizing antibodies. However, viral spread within a host likely relies primarily on direct cell-to-cell transmission [29–32], a process resistant to neutralizing antibodies [33] regardless of the donor’s antibody repertoire [34]. Consequently, while classical neutralizing antibodies may help prevent transmission between individuals, they may be less effective in limiting viral spread within an infected host. This aligns with clinical trials of a subunit gB vaccine, where protection correlated with antibody levels, yet the induced antibodies did not exhibit strong neutralizing activity [25,35]. HCMV infection can also elicit antibodies that mediate antibody-dependent cell-mediated cytotoxicity (ADCC), which is discussed in the next section, however it is notable that the specificity of these antibodies are directed at a variety of HCMV proteins that are not the target for neutralizing antibodies [36].

HCMV primary infection and subsequent reactivations induce a distinct phenotype within the memory B cell compartment. Memory B cells play a crucial role in generating antibodies that complement those produced by long-lived plasma cells, particularly in cases of reinfection or superinfection with different viral strains [37]. In transplant recipients, where T cell responses are often delayed, the B cell-mediated humoral response serves as a critical first line of defence against HCMV replication following reactivation. This response is supported by memory T follicular helper cells specific to the pentameric complex and gB, which facilitate the differentiation of B cells into antibody-producing plasma cells [38].

The number of HCMV-specific memory B cells has been shown to correlate with plasma levels of HCMV-specific antibodies in ageing populations [39]. The memory B cell pool is established during primary infection, during which a significant expansion of activated and atypical HCMV-specific memory B cells has been observed, particularly in pregnant women. These atypical memory B cells express high levels of inhibitory receptors and exhibit reduced TNF-α production [40]. Furthermore, the same research group demonstrated that the frequency of activated gB-specific effector B cells is lower than that of tegument protein-specific B cells, potentially explaining the delayed production of gB-specific IgG during primary infection [41].

### (b) Natural killer cells

Natural killer (NK) cells are predominantly innate lymphocytes that detect malignant and virus-infected cells, traditionally in the absence of antigen-specific receptors [42]. They constitute 5−15% of peripheral blood lymphocytes and are also present in tissues such as the liver, spleen and lungs [43]. Within the innate immune system, NK cells act as a first line of defence, possessing cytolytic activity mediated by the degranulation of secretory lysosomes containing perforins and granzymes [44]. Additionally, they can mediate killing via the expression of tumour necrosis factor (TNF) receptor ligands, including Fas/CD95 ligand (FasL/CD95L), TNF and TNF-related apoptosis-inducing ligand (TRAIL), which bind to their corresponding receptors on susceptible target cells [45,46]. NK cells also release inflammatory cytokines such as IFN-γ, TNF-α, GM-CSF, IL-5, IL-10, IL-12, IL-13, IL-15 and IL-18, as well as chemokines including MIP-1α, MIP-1β, IL-8 and CCL5 (RANTES) [47]. Furthermore, antibodies specific to HCMV proteins expressed on HCMV-infected cells can be bound by NK cells expressing Fc-gamma receptors (FcγRs), primarily FcγRIII/CD16, facilitating ADCC [48].

NK cells express a diverse array of activating and inhibitory receptors on their surface. The net balance of signalling pathways propagated by these receptors enables NK cells to coordinate an appropriate response against HCMV-infected host cells [48]. The critical role of NK cells in anti-viral immunity is evident in patients with impaired NK cell function, who exhibit increased susceptibility to HCMV infection [49,50]. Given their role as rapid responders that target HCMV-infected cells through cytokine secretion, cytotoxic molecules and ADCC, it is unsurprising that HCMV has evolved mechanisms to evade NK cell-mediated immunity. These immune evasion strategies include engaging inhibitory receptors, inhibiting Fc receptor signalling, expressing viral proteins that mimic MHC class I molecules and modulating antigen presentation [51].

HCMV infection and long-term persistence profoundly impact the NK cell compartment, altering its composition by expanding, contracting and reshaping the repertoire of NK cells, which is defined by distinct patterns of activating and inhibitory receptor expression. Notably, there is a significant expansion of NK cells expressing the activating receptor CD94/NKG2C during acute infection. This subset remains overrepresented in seroconverted individuals, with the NKG2C^bright^ phenotype found in approximately 50% of HCMV-seropositive donors [52,53]. It should however be noted that about 4% of humans have a NKG2C^−/−^ genotype, but they still demonstrate NK cell subsets with ‘adaptive’ characteristics [54]. While expansion of NKG2C^+^ NK cells is also observed in other acute and chronic infections, such as human immunodeficiency virus (HIV) and Epstein–Barr virus, it is most strongly associated with HCMV co-infection [55]. Indeed, NKG2C^+^ NK cells selectively proliferate in response to HCMV-infected fibroblasts in a manner dependent on the interaction between NKG2C and its ligand, HLA-E [52].

Importantly, NKG2C^+^ NK cells from HCMV-seropositive individuals undergo extensive adaptation, exhibiting dominant expression of the differentiation marker CD57 and killer cell immunoglobulin-like receptors (KIRs) specific for self-MHC class I molecules, while showing reduced expression of NKG2A, NKp30 and NKp46. These characteristics have led to their classification as ‘adaptive NK cells’ [56]. The direct link between HCMV infection and NKG2C^+^ NK cell expansion is further supported by the accumulation of NKG2C^+^CD57^+^ NK cells following HCMV infection or reactivation in both healthy individuals and transplant patients [53]. Additionally, these memory-like NK cells may have a potent anti-leukaemia effect in the haematopoietic stem cell transplantation (HSCT) setting, potentially reducing the risk of relapse in acute myeloid leukaemia (AML) patients [57]. However, the precise mechanisms underlying this protective effect remain unclear, as the specific viral ligands recognized by NKG2C have yet to be identified [58].

NKG2C^+^ NK cells, along with other HCMV-driven adaptive NK cell subsets [59], exhibit substantial heterogeneity, depending on the combination of activating and inhibitory receptors expressed. These cells also undergo epigenetic remodelling, which influences their effector functions. Specifically, adaptive NK cells display enhanced ADCC and increased production of IFN-γ and TNF-α, yet exhibit reduced responsiveness to cytokine stimulation and diminished degranulation toward activated autologous T cells [60,61]. RNA sequencing (RNA-seq) analysis has further revealed increased expression of *KLR* genes and cytolytic molecules, such as *GZMB* and *PRF1*, within adaptive NK cell clusters derived from the peripheral blood of HCMV-seropositive donors [62]. Further research is needed to fully elucidate the therapeutic potential of adaptive NK cells and their application in targeted immunotherapy [59].

Given the critical role of NK cells as rapid responders against HCMV-infected cells—through cytokine secretion, cytotoxic molecule release and ADCC)—it is unsurprising that HCMV has evolved multiple mechanisms to evade NK cell effector activity, which would otherwise compromise viral fitness. These mechanisms include engaging inhibitory NK cell receptors, suppressing ligands for NK cell-activating receptors, inhibiting Fc receptor signalling (ADCC), expressing viral proteins that mimic MHC class I, and modulating HLA-E expression.

HCMV evades NK cell activation and subsequent targeting by downregulating NK cell-activating ligands on the surface of infected cells. NKG2D, a potent activating receptor expressed on all NK cells [63], recognizes stress- and infection-induced ligands such as MICA/B and ULBP1−6. HCMV glycoprotein UL16 prevents the surface expression of ULBP1, ULBP2, ULBP6 and MICB by retaining them within the endoplasmic reticulum (ER) and *cis*-Golgi compartment [64–67]. Similarly, UL142 downregulates MICA and ULBP3 by retaining them in the *cis*-Golgi. Notably, MICA008, a truncated allele, is resistant to UL142-mediated modulation, making it an ‘escape variant’ that provides a selective advantage to NK cells. However, US9 specifically targets MICA008 for proteasomal degradation; in addition UL147A also targets MICA*008 [68] and UL148A targets full-length MICA [69,70].

The US12 gene family (US12–US21) [71] also targets NK cell-activating ligands, adhesion molecules and cytokine receptors [72]. US12 and US13 downregulate ULBP2 and MICB, while US18 and US20 act synergistically to evade multiple cellular pathways by downregulating the NKp30 ligand B7-H6 and the NKG2D ligand MICA via lysosomal degradation. Additionally, US20 further targets MICB and ULBP2 for lysosomal degradation, demonstrated by proteomics and cell surface flow cytometry analysis, thereby inhibiting NK cell activation [72,73].

UL141 interferes with the NK cell-activating receptor DNAM-1 pathway by downregulating its ligands CD155 (poliovirus receptor/nectin-like molecule 5) and CD112 (nectin-2) [74]. UL141 alone is sufficient to sequester the immature form of CD155 in the ER, while it cooperates with US2 to target CD112 for proteasomal degradation [75,76]. Furthermore, UL141 retains the cellular death receptors TRAIL-R1 and TRAIL-R2 in the ER, thereby inhibiting apoptosis and promoting the survival of virus-infected cells [77]. The secreted gpUL4 also interacts with soluble TRAIL preventing its binding to TRAIL-receptor and induction of apoptosis as well as TRAIL on NK cell membranes preventing degranulation [78].

Natural cytotoxicity receptors (NCRs), including NKp46, NKp44 and NKp30, are uniquely expressed on NK cells. NKp30 directly interacts with HCMV pp65, leading to the dissociation of its signalling adaptor, the CD3ζ chain, thereby inhibiting NK cell-mediated killing [73,79].

In addition to protein-based evasion strategies, HCMV also employs non-coding microRNAs to suppress NK cell cytotoxicity. HCMV miR-UL112 downregulates MICB expression [80,81], and together with the host cellular microRNA miR-376a, further suppresses MICB expression [82]. Moreover, HCMV US25-2-3-p modulates the activity of the metalloprotease inhibitor TIMP3, enhancing metalloprotease activity and increasing the shedding of MICA from the cell surface, thereby reducing NK cell activation [83].

HCMV also encodes viral Fcγ receptors (vFcγRs) that interact with the Fc domains of IgG bound to virus-infected cells, antagonizing host Fcγ receptors—specifically FcγRIIIa/CD16 on NK cells. This inhibits NK cell activation and facilitates ADCC evasion [84,85]. To date, four HCMV-encoded glycoproteins have been identified as vFcγRs: gp34 (encoded by RL11), gp68 (encoded by UL119-UL118), gp95 (encoded by RL12) and gpRL13 (encoded by RL13) [86–88].

HCMV disrupts all stages of the MHC class I antigen presentation pathway to evade CD8^+^ T cell recognition, primarily through US6 family members (US2, US3, US6 and US11). However, downregulation of MHC class I or other ‘self’ markers could render infected cells susceptible to NK cell-mediated cytotoxicity via ‘missing-self’ recognition [89]. Consequently, HCMV has evolved decoy molecules to restore or replace MHC class I, engaging inhibitory NK cell receptors to suppress NK cell-mediated cytotoxicity. UL18 serves as a viral MHC class I decoy, binding to the inhibitory receptor leucocyte immunoglobulin-like receptor subfamily B member 1 (LILRB1) with an affinity more than 1000 times greater than that of endogenous MHC class I [90]. Despite its high structural homology to MHC class I, UL18 is not affected by TAP inhibition mediated by US6 [91]. HCMV UL40 modulates the expression of HLA-E, a nonclassical MHC class I molecule that binds to the inhibitory receptor CD94/NKG2A on NK cells, suppressing NK cell-mediated cytotoxicity. The surface expression of HLA-E depends on the binding of a conserved nonamer peptide derived from classical MHC class I molecules. HCMV glycoprotein UL40 carries the same peptide in its leader sequence, thereby upregulating HLA-E expression and promoting immune evasion [92]. HCMV also targets adhesion molecules, such as the CD2/CD58 axis, through the downregulation of CD58 (LFA-3) by UL148. CD58 serves as a ligand for CD2, a co-activating receptor on NK cells that enhances ADCC via its interaction with CD16 [93]. Notably, adaptive NKG2C^+^ NK cells in HCMV-seropositive individuals exhibit substantial upregulation of CD2 [94], and the CD2/CD58 axis has been shown to play a crucial role in the recognition of HCMV-infected cells by NK cells [95]. CD2 expressing Liver resident NK cells have been shown to have an increased ability to control HCMV infection *in vitro* [96].

### (c) CD8^+^ cytotoxic T cells

The CD8^+^ HCMV-specific T cell response is highly antigenically diverse, recognizing a range of HCMV-encoded proteins expressed at different phases of viral replication (immediate-early, early and late). These target a variety of viral proteins with functions including immune evasion, regulation and structural roles. In HCMV-seropositive individuals, HCMV-specific CD8^+^ T cells constitute a significant proportion of the cellular immune response, comprising approximately 5% of total T cells and 10% of memory T cells [97]. However, there is substantial variability in the magnitude of T cell responses between individuals, and the underlying reasons for this remain unclear.

The most comprehensive study to date, examining IFN-γ responses to 213 predicted HCMV open reading frames (ORFs), found that up to 30% of total CD8^+^ T cells in healthy seropositive adults exhibited an IFN-γ response and recognized a median of eight ORFs [97]. CD8^+^ cytotoxic T cells primarily recognize infected cells through antigen presentation via the MHC class I pathway. They exert effector and cytotoxic functions largely through the secretion of cytokines, including IFN-γ and TNF-α, as well as the release of cytotoxic granules containing perforin and granzymes. Additionally, they can eliminate infected cells via Fas/Fas ligand binding, which triggers apoptosis pathways [98]. In transplant patients, multiple studies have demonstrated that the recovery of the CMV-specific CD8^+^ T cell response is essential for protection against CMV disease [99–101].

Immunophenotyping at different stages of HCMV infection, including in long-term memory, reveals a heterogeneous T cell response with distinct transcriptional profiles, effector functions and chemokine receptor expression patterns, which determine localization. The surface expression of CCR7, a chemokine receptor enabling migration to lymph nodes, along with different isoforms of the tyrosine phosphatase CD45 [102], delineates naive T cells (CD45RA^+^ CCR7^+^). Upon antigen encounter, these cells switch from the CD45RA isoform to CD45RO and undergo extensive antigen-driven proliferation of effector cells, and the generation of memory T cells. Based on differential CD45 isoform and CCR7 expression, memory T cells can be classified into central memory T cells (TCM; CD45RA^-^ CCR7^+^), which predominate in secondary lymphoid organs and exhibit high proliferative potential in response to antigen, and effector memory T cells (TEM; CD45RA^−^ CCR7^−^), which localize to peripheral tissues such as the lungs, liver and gut, where they exert effector functions but have lower proliferative capacity. Following resolution of viremia, a subset of TEM cells specific for HCMV can revert to the CD45RA isoform, forming highly differentiated effector T cells (TEMRA; CD45RA^+^ CCR7^−^). This HCMV-epitope-specific T cell population is cytotoxic in nature, expressing IFN-γ, TNF-α, perforin and granzyme B. It effectively controls viremia without undergoing functional exhaustion and can reacquire proliferative and homing potential upon appropriate stimulation [102–107]. HCMV-specific differentiated T cell subsets are also known to express a diverse range of NK cell receptors [108], which also contributes to the functional capacity of these T cells. Consistent with this, studies of stem cell transplant recipients show a correlation between recovery from active HCMV infection and reconstitution of the CD8^+^ T cell pool [109,110]. We have shown that CD8^+^ T cells co-cultured with HCMV-infected autologous fibroblasts are able to control viral dissemination, however there are distinct differences in efficacy between donors that does not seem to be related to T cell frequency [111].

Recently, HCMV-specific tissue-resident memory T cells (TRM) have been identified in tissues such as the skin and mucosa, where they remain sequestered from circulation. However, their role in controlling HCMV infection in non-lymphoid organs of seropositive individuals remains unclear [112]. HCMV-specific T cells appear to be enriched within memory T cell subsets that are more mature and highly differentiated. This is particularly associated with the downregulation of CD27 and CD28, upregulation of CD57 and killer cell lectin-like receptor subfamily G member 1 (KLRG1), and increased expression of effector molecules such as perforin and granzyme B [113].

HCMV is well known for encoding proteins that interfere with nearly every step of the MHC class I processing and presentation pathway to evade CD8^+^ T cell recognition. The US2, US3, US6 and US11 proteins, belonging to the US6 gene family, function in a coordinated yet mechanistically distinct manner to alter the stability, assembly and export of MHC class I. US2 and US11 target MHC class I for ubiquitin-mediated proteasomal degradation, US3 retains MHC class I in the ER and downregulates MHC class II, while US6 inhibits the transporter associated with antigen processing (TAP), blocking the translocation of viral peptides from the cytosol to the ER lumen [114–118].

Additionally, viral miRNAs have been shown to target components of the immune system. For instance, miR-US4.1 downregulates ERAP-1, an aminopeptidase required for peptide trimming prior to MHC class I presentation, thereby reducing the presentation of immunodominant pp65 peptides to CD8^+^ T cells [119,120]. Similarly, miR-UL148D downregulates CCL5 (RANTES), impairing T cell chemoattraction to infected cells [121].

### (d) CD4^+^ T cells

CD4^+^ T cells are traditionally regarded as helper cells; CD4^+^ T cells facilitate CD8^+^ T cell recruitment, and support the migration of naive CD8^+^ T and B cells to draining lymph nodes and enhancing effector cell responses. In addition, CD4^+^ T cells can exert direct anti-viral activity via cytokine production and/or cytotoxicity [122]. They secrete IFN-γ and TNF-α, and some subsets demonstrate cytotoxic capabilities via perforin- and Fas-mediated killing, particularly in response to cytomegalovirus (CMV) infection [123–125]. These cytotoxic CD4^+^ T cells, which have been observed lysing CMV-infected targets *in vitro*, express the transcription factor Hobit (ZNF683) [125].

Initial studies identifying HCMV-specific CD4^+^ T cells used lysates derived from HCMV-infected fibroblasts to stimulate antigen-specific responses [126–129]. However, a comprehensive study that screened peptide pools representing 213 ORFs from HCMV demonstrated that CD4^+^ T cells recognize up to 125 different ORFs. Notably, CD4^+^ T cells preferentially recognized immediate-early (IE) gene products in addition to viral immune evasion proteins, viral tegument proteins and glycoproteins [97]. The recognition of HCMV glycoproteins by CD4^+^ T cells has also been reported in multiple additional studies [130–132].

The functional characterization of these cells has evolved over time. Early studies primarily assessed intracellular cytokine production, particularly IFN-γ, as a marker of HCMV-specific CD4^+^ T cell responses (reviewed in [133]). More recently, our laboratory has demonstrated that autologous HCMV-specific CD4^+^ T cells, identified by the upregulation of activation markers CD40L and 4-1BB, are capable of restricting viral dissemination in monocyte-derived dendritic cells *in vitro* [134].

Due to the difficulty of identifying patients with primary HCMV infection at the time of diagnosis, most studies on CD4^+^ T cell responses during acute primary infection have been conducted in pregnant women or kidney transplant recipients (D^+^R^−^, where the donor is seropositive and the recipient is seronegative) [103,135–137]. In pregnant women, early post-infection CD4^+^ T cell responses are predominantly directed against gB and pp65 [136]. However, these CMV-specific CD4^+^ T cells exhibit lower functional avidity and express higher levels of immune checkpoint proteins, such as PD-1, compared with memory responses in healthy seropositive adults [136,138,139].

Following the initial proliferative response to primary infection, the CD4^+^ T cell population contracts, leaving behind long-lived memory cells of Th1, Tfh, central memory (TCM) and effector memory (TEM) phenotypes. In healthy CMV-seropositive adults, CMV-specific CD4^+^ T cells span the entire spectrum from naive to central memory T cells, with up to 88% displaying an effector memory phenotype [131]. Furthermore, some CMV-specific CD4^+^ T cells secrete IL-10, a suppressive cytokine, or exhibit a Treg-like phenotype [140–143]. These findings suggest a role in immune regulation, potentially mitigating chronic immune activation associated with CMV infection.

Importantly, CMV-specific CD4^+^ T cells also possess cytotoxic capacity, as demonstrated by surrogate markers such as CD107a expression (a marker of degranulation), intracellular detection of perforin and granzymes, and cytotoxicity assays, including chromium release assays [130–132,134,144–146]. These findings suggest that a subset of CMV-specific CD4^+^ T cells is capable of directly eliminating CMV-infected cells, highlighting their role beyond classical helper functions in anti-viral immunity. CD4^+^ T cells can also have direct anti-viral activity: used in co-culture assays with HCMV-infected autologous fibroblasts CD4^+^ T cells are able to control viral dissemination and demonstrate differences in anti-viral capacity which is donor dependent [111].

US2 has been shown to affect the MHC class II processing pathway by binding to MHC class II α-chains and assembled MHC class II α/β/Ii complexes, leading to their degradation [147]. US3 alters MHC class II complex assembly by binding to HLA-DR (but not HLA-DM) proteins before or during the formation of α/β complexes in the ER, thereby preventing invariant chain binding. This mislocalization of complexes to alternative post-Golgi compartments ultimately reduces antigen presentation in US3-expressing cells [117].

Interferon-γ (IFN-γ) upregulates MHC class II molecules in cells that constitutively express MHC class II, such as B cells, dendritic cells and professional antigen-presenting cells (APCs). Additionally, IFN-γ can induce MHC class II expression in cells that do not constitutively express it, such as epithelial cells and fibroblasts, through activation of the MHC class II transactivator gene (CIITA) [148].

The HCMV genome encodes several proteins that modulate the effects of IFN-γ [149] and directly influence CIITA transcription. In Langerhans cells, a subset of dendritic cells, HCMV infection leads to a reduction in constitutive CIITA expression [150]. Further evidence from transfected cell line models demonstrates that CMV downregulates MHC class II expression on the cell surface through CIITA regulation, independent of known CMV class II modulators US2 and US3 [151].

UL23 interacts with the STAT effector molecule N-myc, preventing the proper activation and nuclear translocation of STAT1 homodimers required for IFN-γ signalling [152]. UL31 preferentially binds to the cytosolic DNA sensor cGAS, leading to the inhibition of interferon-associated gene transcription [153]. The tegument protein pp71 interacts with Daxx, a death domain-associated protein, targeting it for degradation and thereby suppressing the induction of downstream anti-viral genes [154–156]. Additionally, pp71 has been shown to negatively regulate the signalling function of STING (Stimulator of Interferon genes) by inhibiting its translocation to the nucleus and preventing the recruitment of accessory proteins to the signalling complex [157].

Collectively, these viral modulations lead to decreased transcription of interferon-γ-associated genes, ultimately resulting in reduced MHC class II expression on the surface of infected cells and an impaired ability to present antigen via the MHC class II antigen presentation pathway.

Antigen presentation via MHC class II is also disrupted by US2, which targets HLA-DRα and HLA-DMα for proteasomal degradation [147]. Additionally, HCMV inhibits IFN-γ-induced MHC class II transcription by interfering with signal transduction in the JAK/STAT pathway [158]. HCMV also expresses cmvIL-10, a homolog of IL-10, which downregulates MHC class II surface expression, leading to immune suppression and impaired dendritic cell function [159].

### (e) Unconventional T cells

Several minor yet functionally important T cell subsets also contribute to the immune response. These unconventional T cells, which collectively account for up to 10% of the total T cell population [160], play a pivotal role in bridging innate and adaptive immunity. This group includes mucosal-associated invariant T (MAIT) cells, invariant natural killer T (iNKT) cells and γδ T cells, all of which express invariant or semi-invariant T cell receptors (TCRs) and recognize non-peptide ligands [161]. Unconventional T cells are often enriched in tissue compartments such as the liver and gut [160], suggesting a key role in controlling HCMV infection and reactivation at these sites. The following sections summarize current evidence regarding the involvement of each of these subsets in the context of HCMV infection, including findings from healthy individuals, congenital infection cases and transplant recipient cohorts.

#### i. Mucosal-associated invariant T (MAIT) cells

MAIT cells exhibit limited TCR diversity and are defined in humans by the expression of the semi-invariant α-chain Vα7.2-Jα33 [162], typically paired with a restricted set of Vβ chains that recognize ligands presented by the MHC class I-related molecule MR1 [161]. It should be noted that about half of human MAIT cells are encoded by TRAV1-2:TRAJ12 and TRAV1-2:TRAJ20 recombinations (Va7.2-Ja12 and Va7.2-Ja20) [163]. MR1 specializes in presenting vitamin B metabolite derivatives produced by bacteria and fungi. While MAIT cells are most abundant in the blood and liver, they are also found at mucosal sites, including the skin, oral cavity, intestines and respiratory tract [164–166]. These cells express high levels of CD8 and the C-type lectin CD161 [167,168], and exhibit both cytotoxic and cytokine-secreting functions upon activation [161,169].

Although no viral ligand for MR1 has been identified, MAIT cells can be activated in a TCR-independent manner by inflammatory cytokines [170,171]. Alterations in MAIT cell frequency, activation and function have been reported in several viral infections, including SARS-CoV-2, influenza, HIV and hepatitis C (reviewed in [172]). Among herpesviruses, varicella-zoster virus (VZV) has been shown to impair MAIT cell responses to bacterial stimuli [173], while HSV-1 and HCMV suppress MR1 expression. Specifically, HSV-1 downregulates MR1 to inhibit TCR-mediated activation [174], and HCMV has been shown to use the immune evasion protein US9 to block bacterially driven, MR1-dependent activation of primary MAIT cells [175].

The role of MAIT cells in HCMV disease remains underexplored. However, two studies in allogeneic haematopoietic stem cell transplantation (allo-HSCT) cohorts—one in adults [176] and one in children [177]—have reported associations between MAIT cell levels and HCMV reactivation. In the adult cohort, patients with low-level reactivation had significantly higher absolute MAIT cell counts at the time of initial reactivation compared with those with high-level viraemia, suggesting that MAIT cell frequency may serve as a predictive marker for severe HCMV reactivation [176]. In the paediatric cohort, MAIT cell recovery was delayed relative to αβ T cells, and the cells exhibited increased exhaustion and impaired function. Interestingly, higher MAIT cell counts were associated with greater HCMV reactivation, a higher incidence of chronic graft-versus-host disease (GvHD), and fewer late bloodstream infections [177].

Given that MAIT cells are targeted by HCMV immune evasion mechanisms, further studies are warranted to clarify their role in solid organ transplantation and in healthy individuals, particularly in controlling HCMV at mucosal and tissue sites.

#### ii. Invariant natural killer T (iNKT) cells

Two main subsets of NKT cells exist in humans. Type I NKT cells (also known as invariant NKT or iNKT cells) express a CD1d-restricted αβ TCR composed of an invariant Vα chain paired with a limited set of Vβ chains [178] and comprise approximately 1% of T cells in the blood and liver. By contrast, Type II NKT cells, which are more abundant in humans, express a more diverse TCR repertoire, enabling recognition of a broader range of lipid antigens [160]. Recent single-cell analyses have shown that iNKT effector lineages parallel those of CD4^+^ T cells: iNKT1 cells produce IFN-γ, iNKT2 cells produce IL-4 and IL-13, and follicular helper iNKT subsets have been identified, particularly in human tonsils [179,180].

Although viral particles typically lack lipid antigens, iNKT cells have been implicated in anti-viral immunity (reviewed in [181]). Notably, HCMV employs immune evasion strategies that modulate CD1d expression [182–184]. In murine CMV (MCMV) models, iNKT cells have been shown to reduce viral titers and enhance memory CD8^+^ T cell responses through IFN-γ production [185–187].

In humans, ageing is associated with a decline in both the number and function of Vα24^+^Vβ11^+^ iNKT cells [188]. Conversely, CMV infection has been linked to increased frequencies of iNKT cells (CD3^+^CD56^+^), elevated CD57 expression, and enhanced polyfunctionality [189,190]. During primary HCMV infection, iNKT cell proportions remain stable, but their absolute numbers increase due to generalized lymphocytosis. These cells also exhibit heightened activation, as indicated by increased expression of CD38 and NKG2A [138].

In allogeneic haematopoietic stem cell transplantation (allo-HSCT) recipients, higher iNKT cell numbers in the graft have been associated with protection against CMV reactivation [191]. A similar protective effect has been observed in kidney transplant recipients, where CD11b^+^ NKT cells correlated with reduced CMV reactivation post-transplantation [192]. Collectively, these findings suggest that CMV infection influences iNKT cell frequency and phenotype, and that iNKT cells may play a protective role in immunocompromised individuals.

#### iii. Gamma delta T (γδT) cells

Expansions of γδ T cell subsets were first reported over 25 years ago in renal transplant patients with CMV disease [193–195]. The critical role of γδ T cells in anti-viral defence is further underscored by observations in αβ T cell-deficient children, who remain protected from HCMV disease [196–198]. Since then, numerous studies have investigated γδ T cell responses in various immunocompromised populations, including solid organ transplant recipients, allogeneic haematopoietic stem cell transplant (allo-HSCT) patients (reviewed in [199]) and infants with congenital CMV infection [200–204].

Human γδ T cells are broadly categorized into two major subsets: Vδ1 and Vδ2. Vδ1^+^ cells, which pair with diverse γ chains, are predominantly located in peripheral tissues such as the gut epithelium and exhibit adaptive-like features; there are also rarer Vδ subsets including Vδ3^+^ and Vδ4^+^ which are uncommon in blood but enriched in tissue sites such as the liver [161]. By contrast, Vδ2⁻ subsets—including Vδ1^+^ and other non-Vδ2^+^ populations—undergo robust expansion during CMV infection, forming a few dominant, long-lived and highly differentiated clones [205,206]. This expansion is also observed in ageing individuals, where CMV-seropositive elderly display increased differentiation of Vδ2⁻ γδ T cells [207–216].

During primary CMV infection, Vδ2⁻ γδ T cells significantly expand and upregulate CD16 and CD38 [138]. These cells mediate ADCC via CD16 and may also contribute to antibody-mediated rejection in transplant settings [217–219]. In addition, CMV-driven expansions include a Vγ9⁻Vδ2^+^ subset with adaptive-like properties, including cytotoxicity and expression of the inflammation-associated chemokine receptor CX3CR1 [220–223].

Further evidence suggests that γδ T cells localize to vascular endothelial cells during active CMV infection [218,224], where they may interact with the endothelial protein C receptor (EPCR)—a ligand that remains unaffected by CMV [225,226]. Further ligands for γδ TCRs have recently been identified including Annexin-A2, which interacts with the rare Vδ3^+^ subset [227], EPHA2 [228] and HLA-DR [229], which both interact with the adaptive Vδ1^+^ TCR. These findings highlight the potential of γδ T cells to contribute to both anti-viral defence and immune pathology. Their role in controlling HCMV in immunocompromised patients, as well as their utility in clinical monitoring, has been comprehensively reviewed [230].

## 3. HCMV immune responses in healthy, older and the immunocompromised

As outlined in the preceding sections, the immune response elicited by HCMV infection in otherwise healthy individuals is multifaceted, involving both innate and adaptive cellular components, as well as secreted immune mediators. In immunocompromised individuals, the establishment of a *de novo* immune response varies by context. In solid organ transplant (SOT) recipients, such responses typically emerge in the setting of high-level HCMV viraemia. By contrast, in haematopoietic stem cell transplant (HSCT) recipients, immune responses are closely linked to the broader reconstitution of the immune system.

It has become increasingly evident that lifelong HCMV carriage imprints a distinct signature on the immune system. This phenomenon has been proposed as a potential biomarker for identifying transplant recipients at increased risk of CMV reactivation, uncontrolled viral replication and associated morbidity and mortality.

Beyond the transplant setting, these immune alterations may have broader implications for older adults. CMV infection has been implicated as a co-morbidity factor that reshapes immune cell composition and function. While most studies have focused on phenotypic and functional responses to HCMV proteins or peptide antigens, relatively little attention has been given to the direct anti-viral activity of these immune cells.

The potential role of CMV in exacerbating age-related health conditions and accelerating immune senescence has garnered increasing interest. Early studies of immune ageing identified a link between CMV seropositivity and increased all-cause mortality in individuals over 70 years of age—a phenomenon termed the ‘immune risk phenotype’ (IRP) [231]. This association has been observed across diverse populations, with several studies reporting a particularly elevated risk of cardiovascular disease (CVD)-related mortality among CMV-seropositive older adults [8]. This meta-analysis of the association of CMV with risk of CVD mortality included studies where factors such as smoking and other medical co-morbidities were adjusted for. However, other factors including socio-economic status and deprivation also can have impact on both the increased risk of age-related diseases and increased HCMV sero-positivity [232]. This is illustrated by two recent studies where the examined cohorts (UK Biobank and a Netherlands and Danish cohort studies) were predominantly of higher socio-economic status and did not observe a link between CMV infection and incident of cardiovascular disease [233,234].

In addition to its established role in immune modulation, limited studies have explored whether CMV infection or features of immunosenescence are more prevalent in other age-related conditions, including frailty [235–239], sarcopenia [240,241], type 2 diabetes, dementia (reviewed in [242]), increased susceptibility to novel respiratory infections [243] and certain cancers [244]. These studies have yielded mixed results, often relying on CMV IgG serostatus and, in some cases, quantification of CMV-specific T cell responses to assess associations with geriatric conditions.

Expansions of differentiated HCMV-specific T cells in seropositive individuals are a hallmark of CMV-driven immune remodelling and were among the defining features of the IRP. We previously reviewed the often-contradictory findings regarding HCMV-specific T cell expansions in older adults [245]. Many of these studies assumed that age-associated T cell expansions were detrimental to immune competence and contributed to the adverse outcomes linked to CMV infection in the elderly. However, in our large cohort study of individuals aged 20−80 years, we observed no significant age-related changes in the magnitude of HCMV-specific T cell responses, as measured using overlapping peptide pools from HCMV proteins [246]. Notably, when functional capacity was assessed using an *in vitro* viral dissemination assay (VDA), older donors exhibited impaired control of viral spread [134]. We hypothesize that the observed differences in cytokine responses—elicited by peptides via antigen-presenting cells compared with those induced in virus-derived assays utilizing co-cultures of autologous, HCMV-infected cells—are influenced by the immune evasion strategies of HCMV. These strategies include interference with antigen processing and presentation, downregulation of stress-induced ligands for activating receptors, modulation of co-stimulatory signals and enhancement of inhibitory signalling pathways, such as those mediated by immune checkpoint receptors. The net effect of these mechanisms may determine the efficacy of HCMV immune control, which can vary significantly between individuals and may be particularly impaired in older adults.

Building on these findings, we conducted a functional study in an ageing cohort using both viral dissemination and neutralization assays. This revealed that older donors were significantly less capable of controlling viral dissemination and infection compared with younger individuals [111]. Importantly, participants in this functional study had also taken part in the earlier observational study, highlighting that the magnitude of cytokine-secreting CMV-specific T cells did not correlate with functional anti-viral control in this cohort.

A key limitation in ageing research is the frequent exclusion of individuals with multimorbidity or complex medical histories. Many studies are conducted in either healthy ageing cohorts or single-condition groups, limiting their generalizability. This issue was highlighted in the UK Chief Medical Officer’s 2023 Annual Report, *Health in an Ageing Society*, which emphasized that the number of chronic conditions increases with age and that these conditions often interact, compounding symptoms and reducing quality of life [247]. Clinical care, however, remains largely focused on individual diseases rather than the cumulative burden of multimorbidity. This may partly explain the inconsistent associations observed between CMV infection and chronic diseases of ageing.

Although the pathological implications of CMV in age-related diseases remain underexplored, its association with adverse cardiovascular outcomes has prompted more detailed investigation. In a Spanish cohort of patients with chronic heart failure, CMV-seropositive individuals exhibited elevated inflammatory cytokine levels and worse functional status [248]. Similarly, in a large UK cohort of patients with ST-elevation myocardial infarction (STEMI), CMV seropositivity was associated with adverse left ventricular remodelling 12 weeks post-infarction [249].

In our ageing cohort, we also assessed the presence of HCMV DNA at peripheral tissue sites using saliva and urine samples. HCMV DNA was detected in the saliva of two out of nine older donors [111], both of whom exhibited poor PBMC-mediated control of viral spread in functional assays. This observation, along with previous reports of HCMV DNA detection in the urine [250] and blood [251] of older—but not younger—individuals, suggests that reduced immune control may permit low-level peripheral HCMV replication in the elderly. As a result, a greater proportion of cellular and humoral immune resources may be diverted towards controlling CMV, potentially at the expense of responses to other chronic diseases.

Currently, we are investigating whether the immune defect in the control of HCMV infection observed in the older donor cohort is attributable to the expression of inhibitory receptors, potentially in conjunction with a lack of appropriate co-stimulation. This may result from the loss of co-stimulatory receptors or alterations in the specificity of the immune response. Functional viral dissemination assays and neutralization assays, alongside the assessment of reactivating viral DNA in both peripheral tissues and blood, need to be conducted in unwell older cohorts, including individuals with multiple chronic conditions.

A plausible hypothesis is that the functional defect in controlling HCMV infection is more pronounced in these unwell older cohorts, with a corresponding increase in the detection of HCMV DNA in peripheral tissue sites. Should future studies confirm that elderly individuals with multiple chronic diseases exhibit impaired *in vivo* control of HCMV, this would support the testing of therapeutic interventions aimed at enhancing the HCMV-specific immune response and improve overall quality of life. The findings from studies in healthy older individuals provide a valuable baseline for understanding CMV immune functionality in this population and may inform therapeutic strategies applicable to other clinical contexts, such as SOT recipients.

A major objective for clinicians managing patients affected by primary CMV infection or uncontrolled HCMV replication following reactivation is to identify individuals at risk of CMV-mediated disease and adverse effects from current therapies. Several diagnostic methods are currently available, including quantification of antibody levels and avidity, enumeration of CMV-specific T cells, and detection of reactivating viral DNA. The primary diagnostic approach for identifying both primary and past HCMV infection is serology, which detects HCMV-specific antibodies. Typically, the antigen used to screen for HCMV-specific IgG is derived from lysates of fibroblasts infected with the laboratory-adapted HCMV strain AD169. Some commercial and in-house ELISA assays also employ recombinant CMV antigens targeting specific membrane-associated proteins [252]. This distinction is critical, as AD169 lacks expression of the ULb’ gene segment which includes components of the pentameric complex (UL128, UL130 and UL131) [253]. Diagnostic protocols for identifying primary infection in pregnant women often combine IgM and IgG assays with IgG avidity testing to distinguish between primary infection and reactivation. Incorporating CMV-IgG avidity measurements enhances the predictive value of IgM serology for identifying primary infection and potential vertical transmission to the fetus [254–256]. However, a negative result does not definitively exclude congenital CMV infection [257].

An additional challenge encountered in our research is the occasional discordance between CMV IgG serology results obtained from different laboratories using various commercial or in-house assays. For example, in our recent functional assay study of an ageing cohort, one older participant tested negative for CMV IgG using our laboratory’s commercial assay (based on AD169 antigen), yet was initially identified as HCMV-seropositive by the Cambridge BioResource, which employs a different assay. Notably, this donor exhibited detectable CMV-specific T cells and CMV DNA in saliva [111]. This phenomenon—where HCMV-specific antibodies are undetectable in serum despite the presence of CMV-specific B and T cells—has been reported by other researchers [258,259]. These findings suggest that serological assessment alone may be insufficient for defining prior HCMV infection, particularly in transplant donor screening.

In our ageing cohort study, we also observed discrepancies between serum IgG levels against total CMV antigens, gB and pentameric proteins, and the functional capacity of the serum to neutralize infection *in vitro* [111]. This indicates that functional neutralization assays may provide more informative insights into an individual’s humoral response and their capacity to control infection. However, these assays are labour-intensive, require the generation of clinical virus strains, and are challenging to standardize across laboratories. It has been shown that HCMV seropositivity increases for every 10 years of life [260,261]. One can speculate that this is due to chance encounters with the virus over time. In addition, as people get older and circumstances change, e.g. becoming sexually active, changing partners, having children and assuming childcare activities (grandparents in later life), the chance of exposure to HCMV is also changing. It could also be envisaged that as people age and there is an age-related impact on their immune systems, susceptibility to HCMV encounters causing a full primary infection that was previously prevented could also be a potential mechanism. There is not space here to fully discuss the interaction between ageing and HCMV infection, however, a more in-depth discussion of this topic can be found in a special issue of Geroscience (Vol. 39, Issue 3 (Proceedings of the 6th International CMV & Immunosenescence Workshop), pp. 245−303, June 2017).

Although several studies have validated the use of *ex vivo* quantification of CMV-specific CD4^+^ and CD8^+^ T cells—using commercial assays such as QuantiFERON-CMV or intracellular cytokine flow cytometry—to predict significant CMV events [262,263], others have reported limitations. For instance, the QuantiFERON-CMV assay has failed to detect CMV-specific T cells in some cases [264]. Notable phenotypic changes in unconventional T cell populations due to CMV infection in SOT and HSCT patients have been proposed as predictive markers of viraemia [230], including reduced MAIT cell counts in allo-HSCT patients correlating with high viraemic episodes [176]. However, these cellular assays only indicate the presence or absence of a response and do not assess the functional efficacy of these cells in controlling CMV reactivation. Therefore, monitoring CMV viraemia remains essential.

We have also employed functional *in vitro* viral dissemination assays to evaluate the efficacy of the HCMV-specific immune response in a cohort of SOT patients. Our findings demonstrated that enumeration of CMV-specific T cells using HCMV peptide pools did not predict viraemic status [265]. The next step involves applying fully autologous immune cell viral dissemination assays to retrospectively compare anti-viral effector function in viraemic versus non-viraemic patients over time. This approach may ultimately support the development of a real-time assay system to identify patients at risk of viraemia and guide anti-viral intervention strategies.

## 4. Conclusions

Our experience with both ageing and transplant cohorts, as well as insights from other published studies across various clinical settings, indicates that widely used clinical diagnostic tests for CMV can be fallible. When evaluating patients who may be affected by CMV reactivation or whose underlying conditions may be exacerbated by CMV infection, a multifaceted diagnostic approach is essential to comprehensively assess the role of CMV in each case.

It is also critical to continue advancing our understanding of the complex innate and adaptive immune responses in healthy CMV-infected individuals. This foundational knowledge is necessary to interpret the results of functional studies across diverse patient populations and to draw meaningful comparisons.

In complex patient populations, it is imperative to understand the functional consequences of HCMV-driven alterations in the immune response. The magnitude of HCMV-specific antibodies or T cells does not always correlate with an individual’s capacity to control CMV disease. Effective control of HCMV infection depends on a finely tuned interplay between multiple components of the immune system. Therefore, integrating insights from diverse studies is essential to fully elucidate the mechanisms of immune control and to design effective intervention strategies aimed at mitigating both the direct and indirect effects of CMV infection.

## Data Availability

This article has no additional data.
